# Performances of machine learning algorithms for mapping fractional cover of an invasive plant species in a dryland ecosystem

**DOI:** 10.1002/ece3.4919

**Published:** 2019-02-12

**Authors:** Hailu Shiferaw, Woldeamlak Bewket, Sandra Eckert

**Affiliations:** ^1^ Water and Land Resource Centre Addis Ababa University Addis Ababa Ethiopia; ^2^ Department of Geography and Environmental Studies Addis Ababa University Addis Ababa Ethiopia; ^3^ Centre for Development and Environment University of Bern Bern Switzerland

**Keywords:** Afar Region, dryland ecosystems, Ethiopia, fractional cover mapping, invasive alien plant species, machine learning algorithms, *Prosopis juliflora*

## Abstract

In recent years, an increasing number of distribution maps of invasive alien plant species (IAPS) have been published using different machine learning algorithms (MLAs). However, for designing spatially explicit management strategies, distribution maps should include information on the local cover/abundance of the IAPS. This study compares the performances of five MLAs: gradient boosting machine in two different implementations, random forest, support vector machine and deep learning neural network, one ensemble model and a generalized linear model; thereby identifying the best‐performing ones in mapping the fractional cover/abundance and distribution of IPAS, in this case called *Prosopis juliflora (SW. DC.)*. Field level *Prosopis* cover and spatial datasets of seventeen biophysical and anthropogenic variables were collected, processed, and used to train and validate the algorithms so as to generate fractional cover maps of *Prosopis* in the dryland ecosystem of the Afar Region, Ethiopia. Out of the seven tested algorithms, random forest performed the best with an accuracy of 92% and sensitivity and specificity >0.89. The next best‐performing algorithms were the ensemble model and gradient boosting machine with an accuracy of 89% and 88%, respectively. The other tested algorithms achieved comparably low performances. The strong explanatory variables for *Prosopis* distributions in all models were NDVI, elevation, distance to villages and distance to rivers; rainfall, temperature, near‐infrared and red reflectance, whereas topographic variables, except for elevation, did not contribute much to the current distribution of *Prosopis*. According to the random forest model, a total of 1.173 million ha (12.33% of the study region) was found to be invaded by *Prosopis* to varying degrees of cover. Our findings demonstrate that MLAs can be successfully used to develop fractional cover maps of plant species, particularly IAPS so as to design targeted and spatially explicit management strategies.

## 1 INTRODUCTION

In the last 20 years, many studies have attempted to accurately detect the spatial extent of invasive alien plant species (IAPS) to map their spread over time or model their potential invasion area. They have used a variety of environmental, bioclimatic, and/or earth observation data, and applying classification or regression methods. More recently, machine learning algorithms (MLAs) have gained high popularity in ecology and earth science because of their ability to model highly dimensional and non‐linear data with complex interactions and deal with data gaps (Thessen, 2016). Good performances of MLAs have been obtained in several fields, including remote sensing classifications (Mountrakis, Im, & Ogole, 2011) and species distribution modeling (Cutler et al., 2007; Elith & Leathwick, 2009). However, for quantifying the impact of IAPS and developing spatially explicit management strategies, accurate information is crucial not only on the current or projected distribution of IAPS but also on their cover across the invaded range (Le Maitre, Gush, & Dzikiti, 2015; Shackleton, Le Maitre, van Wilgen, & Richardson, 2015a; Shackleton, Le Maitre, van Wilgen, & Richardson, 2015b). A few studies attempted to estimate fractional IAPS cover using remotely sensed data either applying spectral unmixing techniques (Frazier & Wang, 2011; Vilà et al., 2011) or using very high‐resolution remotely sensed data, mostly in combination with machine learning classifiers (Cho, Malahlela, & Ramoelo, 2015; Masocha & Skidmore, 2011). The use of coarser resolution remote sensing resulted in accurate binary maps of presence and absence of IAPS (Chen, Yi, Qin, & Wang, 2017; Wakie, Evangelista, Jarnevich, & Laituri, 2014). Only recently, more promising mapping of IAPS at finer fractions of cover was obtained using a combination of medium or high‐resolution satellite data and powerful machine learning classification algorithms (Ng et al., 2016; Rembold, Leonardi, Ng, Gadain, & Meroni, 2015). Such fine‐scaled and accurate quantification of the local fractional cover of IAPS allows understanding their impacts through cover‐impact curve analysis. Furthermore, it allows to identify areas with early stages of invasion where the control of satellite populations maybe halted or at least slow down further spread of IAPS (Vilà et al., 2011).

*Prosopis juliflora* (Swartz DC.), hereafter referred to as *Prosopis,* has been introduced to different parts of the world with the aim of providing benefits to rural people, such as the production of fuelwood, charcoal, or construction material (Engda, 2009; Haji & Mohammed, 2013; Mureriwa, Adam, Sahu, & Tesfamichael, 2016; Pasiecznik & Henry Doubleday Research Association, 2001). Like numerous other introduced plants, *Prosopis* has become invasive in many places and is increasingly known for its negative ecological and socio‐economic impacts (Shackleton, Le Maitre, van Wilgen et al., 2015a; Shackleton, Le Maitre, van Wilgen et al., 2015b; van Wilgen & Wannenburgh, 2016). In Ethiopia, several studies have attempted to assess *Prosopis* distribution particularly in the Afar Region (Ayanu et al., 2014; Engda, 2009; Wakie et al., 2014), but they either focused on relatively small study areas or provided only coarse‐resolution maps of either presence or absence of the species. Yet, at the early stage of its invasion, or at the invasion front, *Prosopis* often occurs in a patchy mixture with natural vegetation or as single trees, which is challenging to capture by remotely sensed data of moderate spatial resolution. Hence, the development of effective management strategies to mitigate the negative impacts of *Prosopis* requires accurate and detailed information on both invaded areas and on the level of invasion across the invaded area.

We set out to compare the performances of five MLAs (gradient boosting machine implemented in two different ways, random forest, support vector machine, and deep learning neural network), an ensemble model and a generalized linear model. This analysis helps identifying the best‐performing algorithm in mapping detailed fractional cover of *Prosopis* in the dryland ecosystem of the Afar Region, Ethiopia. All model outputs were validated using a number of performance measures. The best‐performing model was then used to create a *Prosopis* distribution and fractional cover map.

## 2 METHODS

### 2.1 Study area and study species

**Figure 1:**
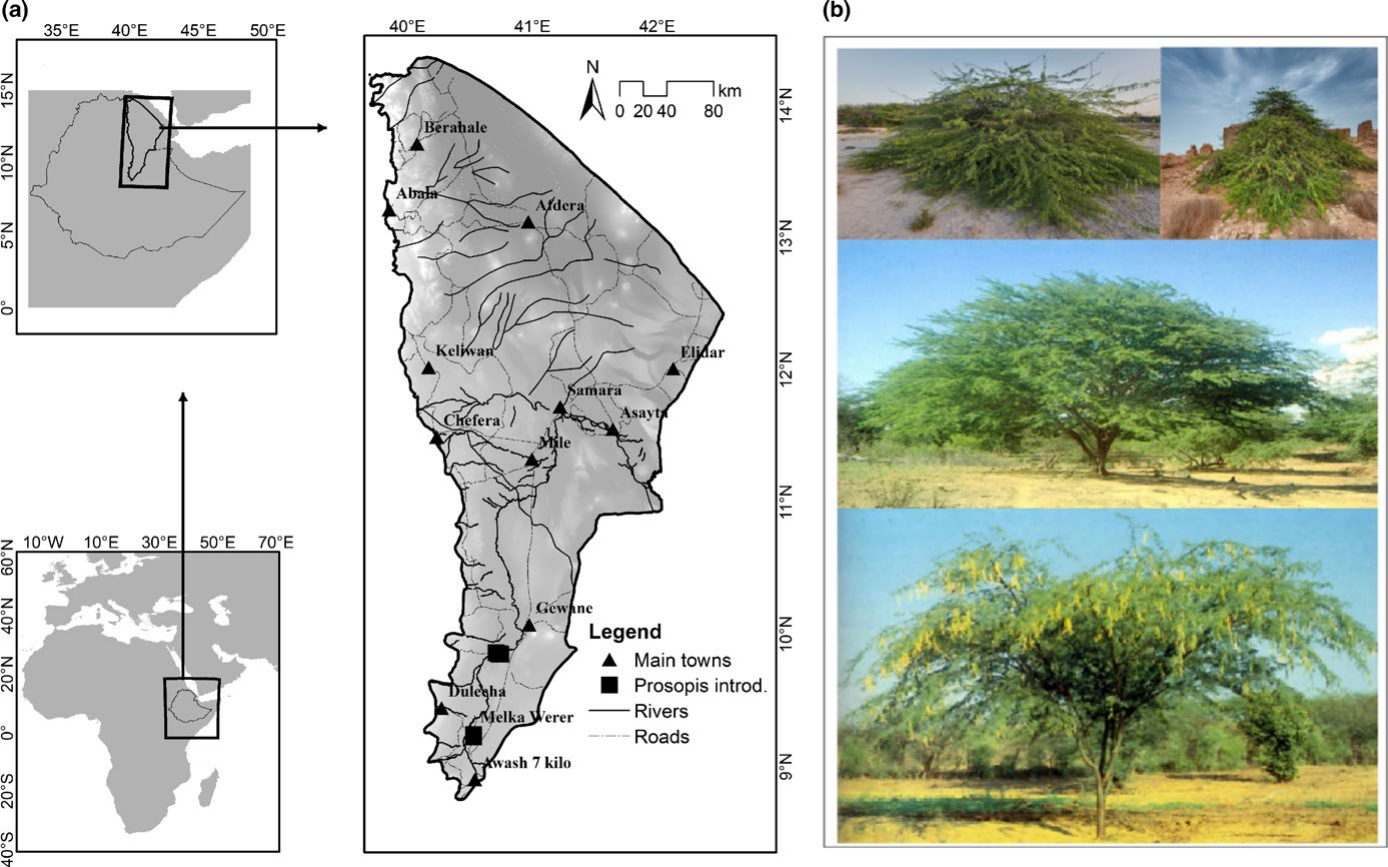
Location of the study area, Afar National Regional State, in Ethiopia (a). The detailed map shows the main towns, roads, and rivers, as well as the locations where *Prosopis* was first introduced. The shading indicates elevation, ranging from 175 m below sea level (dark gray) to 2,992 m above sea level (white), and photos of *Prosopis* plant (b)

The study was conducted in the Afar National Regional State of Ethiopia (hereafter referred to as the Afar Region). The study area extends from 39.7°E to 42.4°E and 8.8°N to 14.5°N, and is located in the Great Rift Valley of Eastern Africa and covers an area of 9.51 million ha (Figure 1a). Mean annual rainfall is about 560 mm; and the mean annual temperature is about 31°C (MOA, 1997). The biome can be described as semi‐arid to arid. Its vegetation cover consists of patches of scattered dry shrubs, acacia woodland (comprising different *Vachellia* species), bushland, grassland, and wooded grassland. People's main sources of livelihood are pastoralism and some agro‐pastoralism around small rural towns (Yirgalem, 2001).

The study focuses on *Prosopis* species. *Prosopis* shows a wide range of ecological adaptations (from arid to tropical climate conditions) and occur along a large variety of environmental gradients (Asfaw & Thulin, 1989; Mohamed, 1997), including different soil types (from sand to heavy clays and stony soils) and a wide range of altitudes (from sea level up to 1,600 m. a.s.l: Shiferaw et al., 2019). Furthermore, *Prosopis* trees are able to fix nitrogen and have deep root systems, rendering them resistant to droughts (Keller, Lodge, Lewis, & Shogren, 2009; Mohamed, 1997). This has enabled *Prosopis* to become one of the most successful invasive woody plant species in arid and semi‐arid areas. *Prosopis* has been planted to reclaim degraded land, combat desertification, reduce soil erosion (Mishra, Crews, & Okin, 2014; Pasiecznik & Henry Doubleday Research Association, 2001; Tessema, 2012; Wakie, Evangelista, & Laituri, 2012), and manage soil salinity (El‐Keblawy & Al‐Rawai, 2007). *Prosopis* trees originally planted in Ethiopia (Figure 1a) belong to the species *P. juliflora* (Figure 1b) in the late 1970s and early 1980s with the main aim of soil and water conservation (Pasiecznik & Henry Doubleday Research Association, 2001). However, since the early 1990s, its invasive nature has caused major problems in rangelands, agricultural fields, and riverbanks, and aggravating conflicts on grazing land among pastoralists (Argaw, 2015; Kebede & Coppock, 2015; Tegegn, 2008). Such conflicts have been common in the Awash Basin, where *Prosopis* has invaded vast areas of precious rangeland and cropland (Wakie et al., 2012).

### 2.2 Sampling design and datasets

Georeferenced field samples were collected throughout the entire study area using a stratified random sampling approach. Presence and absence plots were selected from invaded and uninvaded areas, respectively. Invaded areas were additionally stratified into heavily invaded and less invaded areas. Within those strata, careful attention was paid to collect representative samples of the entire cover gradient (0%–100%) of *Prosopis* coverage. In order to reduce spatial autocorrelation, each sampling plot had a minimum distance of 500 m to the next one. A total of 2,722 samples (presence and absence plots of 20 m × 20 m) were collected between September 2016 and March 2017. A plot was considered a presence plot if it contained at least one *Prosopis* plant; otherwise, it was considered an absence plot. About 70% of the samples were absence plots while 30% were presence plots. These shares were chosen based on a preliminary rough estimation of the shares of uninvaded and invaded land in the study area, which would avoid any bias of results toward either presence or absence of *Prosopis* (Jiménez‐Valverde & Lobo, 2007). Finally, 80% of all sampling plots were randomly selected to be used for model calibration, whereas the other 20% were used for validation (Elith et al., 2011).

The spatial datasets were gathered from various sources and used as explanatory variables to run the models (Table 1). Explanatory variables differed in terms of spatial resolution, projection, and time of acquisition; thus, reprojection to UTM projection and nearest neighbor spatial resampling to a pixel resolution of 15 m was applied using panchromatic band of Landsat 8. The Landsat 8 (operational land imager‐OLI) satellite data were acquired on 26 and 28 January as well as 11 and 20 February 2017 (paths: 167 and 168; rows: 50–54). In total, nine scenes were required to cover the entire study area and then mosaicked. These acquisition dates match the period of field data collection and fall into the study area's dry season, when herbs and grasses are dry and most trees and bushes except *Prosopis* have shed their leaves.

**Table 1:** List of spatial data and explanatory variables used for the modeling of *Prosopis* fractional cover

Variable abbreviations	Description	Source
Rain	Mean annual rainfall	Ethiopian National Meteorol. Agency
Temp	Mean monthly temperature	
LSTd	Monthly land surface temperature during daytime and nighttime; for the modeling 5‐year averages were calculated	MODIS, NASA
LSTn	Monthly land surface temperature during nighttime; for the modeling 5‐year averages were calculated	MODIS, NASA
PAN	Panchromatic reflectance	Landsat 8 OLI, USGS
Red	Red reflectance	Landsat 8 OLI, USGS
NIR	Near‐infrared reflectance	Landsat 8 OLI, USGS
SWIR1	Shortwave‐infrared band 6 reflectance	Landsat 8 OLI, USGS
NDVI	Normalized difference vegetation index	
Elevation	Shuttle Radar Topography Mission digital elevation model (30 m spatial resolution)	USGS
Slope	Derived from elevation	
Relief	Derived from elevation (contour) differences	Adediran, Parcharidis, Poscolieric, and Pavlopoulos (2004)
Landform	Topographic position index derived from elevation, aspect and slope	Dikau (1989); Dikau, Brabb, & Mark (1991); Weiss (2001); Ilia, Rozos, & Koumantakis (2013)
Rugged	An index derived from elevation	Riley, DeGloria, & Elliot (1999)
DistRoad	Distances derived from road network data	Ethiopian Road Authority
DistVillage	Distances derived from settlement data	EthioGIS and Central Statistical Agency
DistRiver	Distances derived from data on watercourses	EthioGIS

The remotely sensed datasets were checked for geometric correspondence to all other datasets. Further, these datasets were atmospherically corrected using the Landsat Ecosystem Disturbance Adaptive Processing System (LEDAPS) algorithm (Chavez, 1996; Lu, Mausel, Brondizio, & Moran, 2002). The Red, the near‐infrared (NIR) and the first shortwave‐infrared (SWIR1) bands of Landsat 8 were selected as explanatory variables. Furthermore, the normalized difference vegetation index (NDVI) was calculated from Red and NIR bands and used as another input. All selected bands, as well as the NDVI, have proven to be particularly suitable to capture photosynthetic active vegetation, and soil and vegetation moisture content (Barsi, Lee, Kvaran, Markham, & Pedelty, 2014). Additionally, daytime (LSTd) and nighttime (LSTn) land surface temperatures from MODIS sensor were included. In order to have longer‐term LSTd and LSTn average data, a 5‐year average of these products was generated between 2012 and 2017. The spatial resolution of these datasets is 1 km. Although this seems rather low compared to the other datasets, these day‐ and nighttime temperature datasets have shown to be useful in species distribution modeling and, particularly in Africa where weather stations are scarce. These datasets have shown to be more accurate than other global climate datasets (Ashby, Moreno‐Madriñán, Yiannoutsos, & Stanforth, 2017; He et al., 2015). Moreover, we used variables representing topography, infrastructure as well as watercourses as these variables have shown to have an influence on *Prosopis* distribution (Shiferaw et al., 2019).

### 2.3 Models

Our study evaluates the performances of seven algorithms in mapping *Prosopis* distribution and fractional cover abundance. We chose five MLAs: two different implementations of gradient boosting machine (GBM and GBM‐BRT), random forest (RF), support vector machine (SVM), and deep (learning) neural network (DNN), an ensemble model composed of the four best‐performing tested algorithms, and a generalized linear model (GLM) for comparison reasons. All model calculations and model performance assessments were implemented in R programming (R Core Team, 2017). A comprehensive overview of the R packages and the different parameter settings are provided in the Supporting information (Table S1). We checked collinearity of explanatory variables before applying to any model and those having high variable inflated factors (VIF) were removed. In this study, we used a threshold level of VIF > 10 to exclude variable(s) from any model (Bruce & Bruce, 2017; Gareth, Witten, Hastie, & Tibshirani, 2014). Accordingly, three variables, the blue, green and second shortwave‐infrared (SWIR 2) bands were removed from all models as they had high VIF (Dormann et al., 2012). We then assessed the influence (importance) of variables in each model by using the method described by Natekin and Knoll (2013). Furthermore, 10‐fold cross‐validation was applied to assess model performance (Fushiki, 2009). Finally, the predictive power of all tested MLAs was evaluated using several performance parameters (Table 2). The general functionality of each tested model is described below.

**Table 2:** Parameters used to assess model performance

Perf ormance parameter	Description	Sources
Confidence interval (CI)	It provides a range of values within which the population parameter is likely to lie. In a normal distribution, the general expression of the confidence interval is: Estimate ± $\frac{Z\alpha }{2}$(*SE*), where *SE* is the standard error of the estimate and, if α = 0.05, *z* = 1.96. The provision of confidence limits in addition to accuracy is particularly useful in comparative analyses	Newcombe (1998)
Correlation	Agreement between fractional cover measured in the field samples and the predicted fractional cover for the same samples	Harrington (2006); Meynard & Quinn, (2007)
Sensitivity	Known as true‐positive rate (TPR); measures the proportion of positives that were correctly identified as locations where *Prosopis* was present. Calculated as: $\frac{\text{TP}}{\left(\text{TP}+\text{FN}\right)}$; where TP stands for true positives, and FN for false negatives	Metz (1978); Fuchs, DeMeester and Albertucci (1987)
Specificity	Known as true‐negative rate (TNR); measures the proportion of negatives that were correctly identified as locations where *Prosopis* was absent. Calculated as:$\frac{\text{TN}}{\left(\text{TN}+\text{FP}\right)}$;where TN stands for true negative, and FP for false positives	Fuchs et al. (1987)
Accuracy	Class accuracy is calculated by dividing the number of correct pixels in that category by the total number of pixels in either the corresponding row or the corresponding column; it indicates the probability of a reference pixel being correctly classified and is really a measure of omission error. Calculated as: $\frac{\text{TP}+\text{TN}}{\left(\text{TP}+\text{FP}+\text{TN}+\text{FP}\right)}$; where TP stands for true positives, TN for true negatives, FP for false positives, and FN for false negatives	Congalton (1991) Fuchs et al.(1987)
AUC	Area under the receiver operating characteristics (ROC) curve; indicates the model's accuracy in handling true values (presence of *Prosopis*) as true and false values (absence of *Prosopis*) as false. The higher the AUC, the better the model fit, and vice versa	Landis & Koch (1977); Metz (1978)
Kappa coefficient	Statistical measure of inter‐rater agreement, excluding agreements occurring by chance. It is calculated in a confusion matrix as $\frac{\left(0.5\times \text{TP}\right)}{\left(\text{TP}+\text{FN}\right)}+\frac{\left(0.5\times \text{TN}\right)}{\left(\text{TN}+\text{FP}\right)}$	Metz (1978)
Balanced accuracy	Average of all class accuracies; takes into account unbalanced class sizes. In our case, with two classes (presence and absence of *Prosopis*) it is calculated as: $\frac{1}{2}\left(\frac{\text{TP}}{P}+\frac{\text{TN}}{N}\right)$	Brodersen, Ong, Stephan, and Buhmann (2010)
Threshold (max @ TPR + TNR)	Maximum value at which the true‐positive rate (TPR, or sensitivity) and the true‐negative rate (TNR, or specificity) intersect. It is often used as a threshold level in dichotomies. In our case, values above the threshold indicate that *Prosopis* is present; values below the threshold indicate that *Prosopis* is absent	Metz (1978); Getis & Ord (1992); Hijmans and Elith (2015)

Until few years ago, multivariate linear regression was the most commonly used approach in species distribution modeling (Collingham, Wadsworth, Huntley, & Hulme, 2000; Higgins et al., 2003; Stohlgren et al., 2010). In this study, the GLM was included to compare the performance with the MLAs (Nicholls, 1989; Getis & Ord, 1992). We used backward and forward stepwise variable selection to find a parsimonious model (Pearce and Ferrier, 2000). Akaike Information Criterion was used as the model performance metric (step‐AIC; Higgins et al., 2003).

Gradient boosting machine as well as GBM‐BRT use a boosting approach where datasets are resampled several times to generate results that form a weighted average of the resampled dataset. This is done by creating a gradient (or step‐by‐step) boosting by minimizing errors among series of decision trees that together form a single predictive model (Natekin & Knoll, 2013; Olinsky, Kennedy, & Kennedy, 2012; Wana & Beierkuhnlein, 2010; Boser,Guyon, & Vapnik, 1992). In our study, we tested two implementations of GBM and GBM‐BRT. They are both based on the same packages: “gbm,” “caret,” “dismo,” and “raster,” with “dismo” and “caret” using the “gbm” package to fit the models. The main differences of the two implementations are the use of different hyper‐parameters. We varied the interaction depth (i.e., tree complexity in GBM‐BRT) which we set to 3 for GBM and was set to 5 for GBM‐BRT, as well as the loss function. While GBM used the “Gaussian” family (Friedman, 2001), GBM‐BRT used the "Bernoulli" (Elith, Leathwick, & Hastie, 2008). Furthermore, the final selection of number of trees and the learning rate was different. We tuned the models by only varying the number of trees and the number of repeats while other parameters were kept stable using their respective R package default settings (for details see also Supporting information Table S1). Fine‐tuning the number of iterations is done to improve the performance of a model by fitting either many sub‐models or gradient fitting and combining them for final prediction. All models were tuned using the same performance metrics. For the fine‐tuning, we calculated mean change in predictive deviance ±one standard error (Elith et al., 2011). The optimization of the number of trees improved the performance substantially (Supporting information Figure S1).

The RF builds the trees in parallel processes (Breiman, 2001). The trees are fully grown and each is used to predict the out‐of‐bag observations that do not occur in a bootstrap sample (Breiman, 2001). The predicted class of an out‐of‐bag observation is calculated average of the results of all predictions (Breiman, 2001; Youssef, Pourghasemi, Pourtaghi, & Al‐Katheeri, 2016). The RF has some limitations like incapable of predicting beyond the range of response values in the training data (Hengl et al., 2015), and overestimate low values and underestimate high values (Horning, 2010). In this study, we only varied the number of trees, testing two different settings: 1,000 and 5,000 trees while all other parameters were set to default.

The SVM can be used for classification or regression. It constructs a hyperplane or set of hyperplanes in an infinite‐dimensional space and tries to find the optimal separating hyperplanes, that is, the planes where the separability between classes is at its maximum (Noble, 2006; Rodrigues & De la Riva, 2014). The SVMs have many mathematical features that make them attractive for prediction, handle extremely high‐dimensional feature spaces, and identify outliers (Brown et al., 2000; Kimothi & Dasari, 2010). We varied settings for the kernel, the cost function and gamma (Supporting information Table S1).

The DNN has become very popular recently but is still sparsely used by the geoscience community (Zhang, Zhang, & Du, 2016). The DNN is fully connected neural networks composed of multiple hidden layers together with non‐linear transformations and a variety of tailored architectures (Guo et al., 2016). The DNN has a capacity to analyze big data. In this study, we used a feed‐forward neural network.

**Figure 2:**
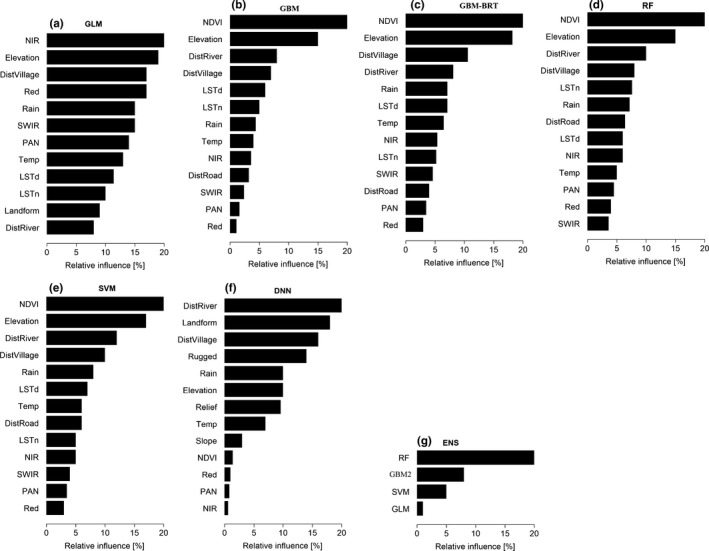
Relative influence of explanatory variables in the different algorithms after removal of the least‐contributing ones: (a) generalized linear model (GLM), (b) gradient boosting machine (GBM), (c) gradient boosting machine using boosted regression trees package (GBM‐BRT), (d) random forest (RF), (e) support vector machine (SVM), (f) deep learning neural network (DNN), (g) ensemble model (ENS)

The purpose of ensemble models is that it should combine the benefits of each included optimized model and penalize the overestimate or underestimate of each individual model. Thus, in order to be able to do so they should be diverse and complement each other on the one hand, but also each one of them independently achieving a high performance (Chitra & Uma, 2010). Our ensemble model consisted of the four best‐performing models (RF, GBM, SVM, and GLM). They were weighted using the function “glmnet” where the predictions from each model are used as a predictor in a GLM and the resulting GLM coefficients determine how much each model should be weighted (Hastie & Qian, 2016; R Core Team, 2017). The coefficients of contribution of each model in our ensemble were 0.2 for RF, 0.1 for GBM, 0.05 for SVM, and 0.01 for GLM as indicated in Figure 2.

## 3 RESULTS

### 3.1 Model parameter settings and weighting of variables

Optimum performance of the GBM‐BRT was found when using ~6,050 trees than 3,100 trees; while the GBM performed better with 500 trees than 100 trees. The RF model performed better for 5,000 trees than with 1,000 trees. The tested algorithms weighted the explanatory variables differently, depending on each model's sensitivity to small variations in the data and to the variable types (Figure 2a–g). In all models, Relief, Landform, Rugged, and Slope were removed again from the model except from the DNN. In the DNN, the least important variables were NIR, PAN, Red, NDVI, and Slope. Interestingly, DistRoad, Rugged, Relief, and Slope proved to be among the least‐contributing variables in the GLM model and were removed from the final iteration (Figure 2a) though DistRoad was one of the important contributors in other models. In the MLAs, 13 out of the 17 variables were kept. The most important explanatory variables, having >5% relative influence, were selected by more than one MLAs. These are NDVI, Elevation, DistVillage, DistRiver, Rain, NIR, Red, LSTd, and LSTn in decreasing order. The first four variables had the highest influence in four of the seven models to explain *Prosopis* distribution (Figure 2).

### 3.2 Evaluation of the models

Among the tested models, the RF performed the best, followed by the ensemble model, GBM and SVM (Table 3). The last two performed comparably. While the GBM achieved slightly higher accuracies and kappa statistics than the SVM, but the SVM obtained better sensitivity and specificity scores. While the GBM‐BRT achieved high accuracy compared to the GLM but its kappa, sensitivity, and specificity scores are low. However, the GLM's specificity score was higher than the ones obtained by the GBM‐BRT model. DNN did not perform well. Its sensitivity and specificity scores were very unbalanced and its sensitivity score was very low. All models performed better in terms of specificity than sensitivity. This indicates that uninvaded areas (true absence rate) were better identified and classified than invaded areas (true presence rate).

**Table 3:** Summarized performance parameters of the evaluation of current fractional cover maps of *Prosopis* in the Afar Region produced by means of different models. Additionally, AUC plots for each model are provided in the Supporting information Figure S2

Model type	95% CI	Accuracy	Kappa	Balanced accuracy	Sensitivity	Specificity	Pos. pred. value	Neg. pred. value	AUC	Correlation	Threshold
GLM	0.763, 0.834	0.801	0.498	0.744	0.612	0.882	0.651	0.858	0.852	0.564	0.285
GBM	0.837, 0.897	0.877	0.678	0.848	0.802	0.895	0.738	0.924	0.944	0.756	0.397
GBM‐BRT	0.761, 0.841	0.789	0.316	0.727	0.632	0.712	0.728	0.868	0.945	0.794	0.258
RF	0.897, 0.945	0.918	0.797	0.911	0.894	0.926	0.818	0.959	0.971	0.829	0.326
SVM	0.856, 0.907	0.872	0.677	0.827	0.817	0.918	0.864	0.891	0.876	0.741	0.151
DNN	0.689, 0.767	0.729	0.434	0.574	0.014	0.995	0.568	0.724	0.595	0.206	0.392
Ensemble	0.871, 0.925	0.891	0.771	0.873	0.846	0.919	0.808	0.939	0.962	0.841	0.349

Note

DNN: deep learning neural network; ENS or ensemble: ensemble model; GBM: gradient boosting machine; GBM‐BRT: gradient boosting machine using boosted regression trees package; GLM: Generalized linear model; RF: random forest; SVM: support vector machine.

### 3.3 *Prosopis* fractional cover

**Figure 3:**
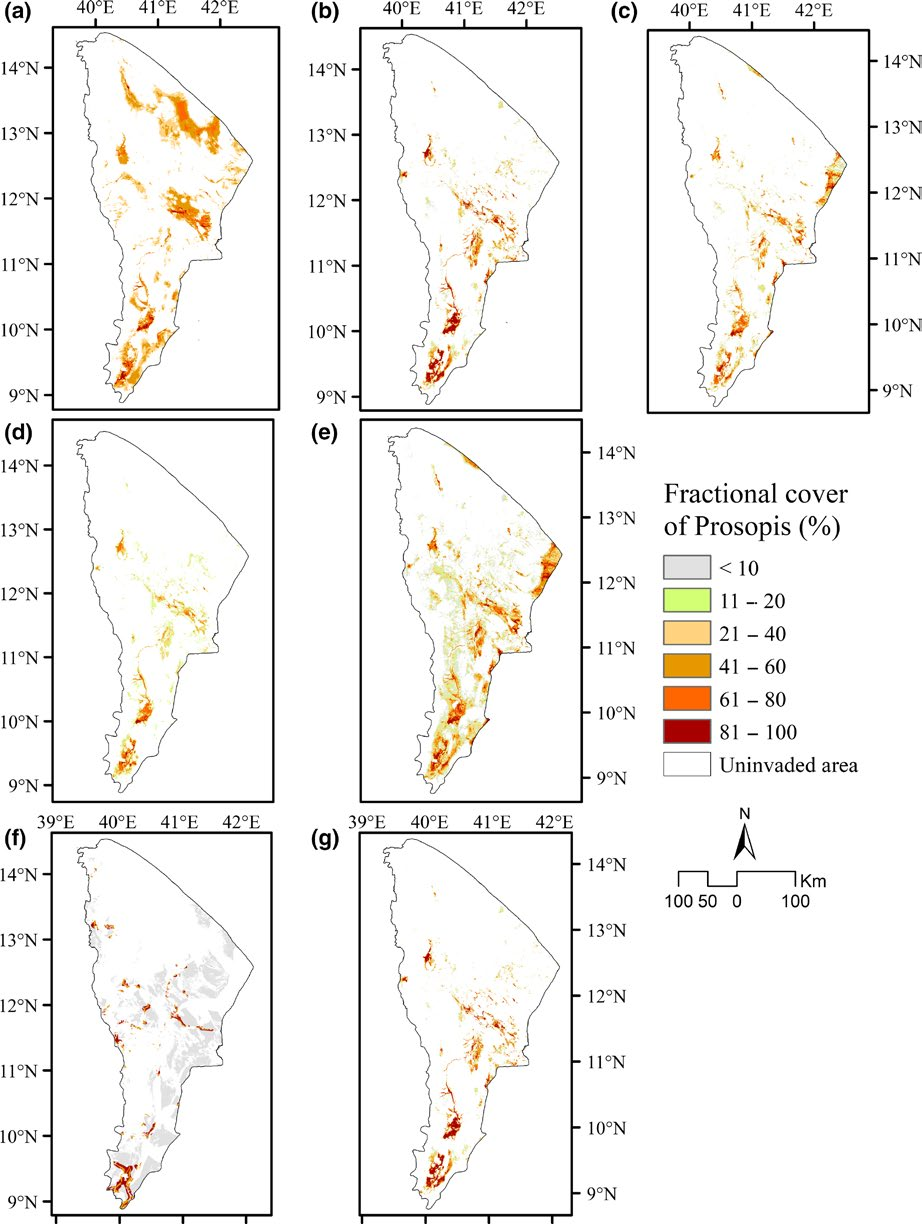
The current fractional cover maps of *Prosopis* distribution were produced by using different machine learning algorithms. (a) generalized linear model (GLM), (b) gradient boosting machine (GBM), (c) gradient boosting machine using boosted regression trees package (GBM‐BRT), (d) random forest (RF), (e) support vector machine (SVM), (f) deep learning neural network (DNN), (g) ensemble model (ENS)

Comparing the results of different models, we found considerable variation in the extent of invaded areas, even though we used the same input datasets for all algorithms. The most extreme estimates of the total area invaded by *Prosopis* were generated; the highest was from the DNN model (34.8% invaded) and the lowest was from the SVM model (11.2% invaded). The best‐performing RF model, calculated the total invaded area to be 12.33% of the Afar Region. The other four models—the GBM‐BRT, the ensemble model, the GBM, and the GLM estimated the total invaded area by *Prosopis* at 16.1%, 14.9%, 14.7%, and 20.1%, respectively (Figure 3 and Table 3). Hence, the results produced by the ensemble model, the SVM, and the GBM were fairly close to that produced by the RF model (Table 3).

**Figure 4:**
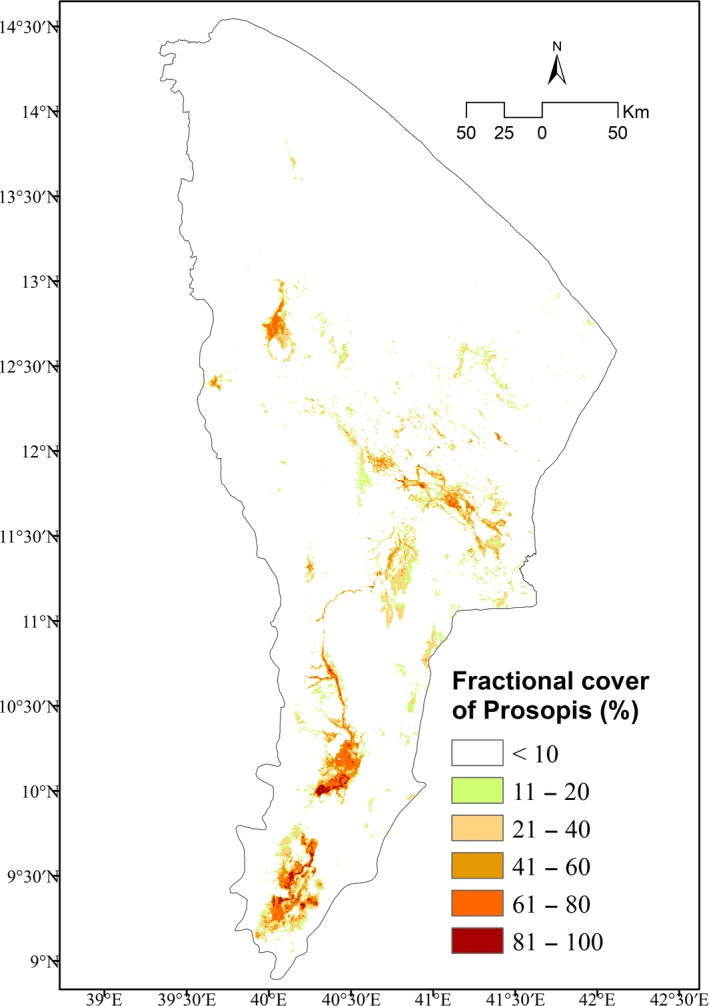
Current fractional cover of *Prosopis* (after matching to the ground cover level) in the Afar Region according to the RF model. For better readability, fractional cover was grouped in six fractional cover classes

Following the evaluation of the different models, the best‐performing RF model was used to map the current fractional cover of *Prosopis* in the Afar Region. The RF model's sensitivity and specificity values suggest that the model is robust, and its AUC value indicates that the presence of *Prosopis* was correctly mapped with a probability of 97%. A threshold value of 0.326 was calculated from the model for the minimum cover level of *Prosopis* presence, which corresponds to 0.4% *Prosopis* fractional cover found on the ground (Figure 4). According to the RF prediction, about 1.173 million ha of land is invaded by *Prosopis* at different stages of cover abundances in the Afar Region.

## 4 DISCUSSION

### 4.1 Model optimization

During model optimization, the number of trees (for the GBM‐BRT, GBM, and RF), the learning rate (sets the weight applied to individual trees), and the bag fraction (which sets the proportion of observations) had the greatest influence on model performance (Elith & Leathwick, 2009). For example, the lower of two learning rates tested in the GBM‐BRT required more trees, which improved the result without causing overfitting (Mining, 2009; Hijmans & Elith, 2013, 2015). Consequently, the lower learning rate of 0.005 with 6,050 trees performed better than that of 0.01 with 3,100 trees. However, a learning rate of 0.0025 with 10,000 trees did not perform better than that of 0.005 in the GBM‐BRT even though the increase in the number of trees reduced deviance, eventually stabilizing the model. This indicates that lowering the learning rate without comparing model performance would have resulted in two disadvantages: a poorer model fit and longer computational time without improving the model's accuracy.

Variable reduction contributed to model stability, which is evident in the GBM‐BRT and GLM models. Similar studies in the GBM showed model stability after variable reduction (Getis & Ord 1992; Burnham & Anderson, 2002). Removal of the topographic variables such as Rugged, Landform, Relief, and Slope from most of the tested models indicates that these variables contributed little to the models’ performances. Also, except through Elevation, topography does not seem to add significant information regarding the current distribution and cover of *Prosopis* in the study area. This is probably because the study area is largely flat. The DNN model produced one of the least accurate results. The ROCs of GLM and DNN showed different from other MLAs (Supporting information Figure S2). The ROC curve in the GLM nears quickly the 100% true positives rate but the ROC curve in the DNN remains flat achieving a comparably high amount of false‐positive rate compared to its true‐positive rate. Different reasons could have led to a poor performance, for example, batch size may be small to the DNN. But then there are things like to check for hidden dimension layers, analyze the gradient checks. Further tuning might have been necessary to improve the DNN (change a different optimizer, change regularization, check and adjust weights at initialization, etc). However, this requires further investigation.

### 4.2 Important variables

Among the infrastructure variables, DistVillage was found to be important in all models except in the GLM. Among the environmental variables, Elevation was the most important explanatory variable for the distribution and fractional cover of *Prosopis* in all models except the DNN. While NDVI and DistRiver had a high relative importance in the MLAs, they were removed from the GLM during variable reduction. From a methodological perspective, this suggests that the GLM is not able to relate variables having a linear or radial spatial pattern to the samples used in the models, and therefore, is less suited to explain *Prosopis* distribution and fractional cover. It is well known that *Prosopis* is primarily spread by livestock (Shiferaw, Teketay, Nemomissa, & Assefa, 2004), human transport and along watercourses, thereby promoting discontinuity or jump dispersal (Wilson, Dormontt, Prentis, Lowe, & Richardson, 2009). However, the GLM was not able to fully capture these phenomena. In the DNN model, Landform exceptionally ranked second in importance, following DistRiver.

The influences of the tested explanatory variables varied in terms of magnitude and direction depending on each model's sensitivity. In the case of NDVI, this is in line with the general observation of greenness, and therefore, also NDVI, increases with increasing *Prosopis* cover. It suggests that particularly NDVI captured during dry season is a good variable for explaining the current distribution of *Prosopis* due to the plant's evergreen behavior in the study area unlike other plant species shed their leaves during the dry season. The explanatory power of NDVI is further supported by the fact that greenness or NDVI is a consequence of *Prosopis* presence and cover level but not a cause of its distribution. Our results also show that *Prosopis* cover increases with increasing temperature. *Prosopis* grows best in arid and semi‐arid environments and can stand air temperatures of up to 50°C (Mohamed, 1997). Besides temperature, elevation had a strong influence on *Prosopis* distribution in the study area as *Prosopis* cover increases with decreasing elevation.

As mentioned above the main causes of dispersal are by livestock, human transport and by water which explains well the strong influence of these factors in most models. In contrast to Menuz & Kettenring (2013), our data suggest that landscape structure variables are more relevant for species distribution/invasion at the current stage of invasion than climatic factors (precipitation and temperature), which describe the environmental niche of plant species (Guisan & Thuiller, 2005). However, at larger spatial scales climatic factors might additionally capture well the distribution pattern of the species (Coutts, Klinkenvan, Yokomizo, & Buckley, 2011; Cabra‐Rivas, Saldana, Castro‐Dıez, & Gallien, 2016).

### 4.3 Fractional cover of *Prosopis*

Different algorithms produced different results with varying accuracies. Thus, these algorithms differ in their sensitivity (power to distinguish *Prosopis* distribution from other vegetation) across spatial variabilities. In this study, we found the RF to be the best‐performing algorithm (AUC = 0.971, *κ* = 0.797). Surprisingly, the ensemble model (AUC = 0.962, and κ = 0.771) performed slightly less than the RF, although other studies had suggested that an ensemble model would be able to overcome some of the individual models’ limitations (Kim, 2017) and expected to obtain better performance. Our finding indicates that some of the models included in the ensemble model might have introduced errors, thereby impairing or penalizing its performance. Also, the DNN did not perform well which we cannot fully explain. Reasons could be the DNN may not be appropriate for species distribution mapping and requires further investigations.

Application of a threshold level to produce binary maps of presence and absence has been tested (Zhou, Chen, Cao, & Chen, 2015). In this study, we also applied threshold levels to distinguish invaded from uninvaded areas with a threshold level of the RF model at 0.326. Based on this threshold, we found a very large area (~1.173 million ha) to be invaded. Our result is in line with the amount of invaded areas estimated by MoLF (2017) to be about 1.2 million ha.

Detection of the spread and establishment of an invasive plant species is highly important for an effective management at an early stage of invasion (i.e., low to medium cover levels). Soft classification, as performed in this study, based on satellite data, climatic, topographic, and other relevant data enables not only identification of a particular species but also retrieval of that species’ fractional cover even at low cover fractions. Another interesting finding is that all models performed better in terms of specificity than sensitivity (Table 3). This indicates that uninvaded areas (true absence rate) were better identified and classified than invaded areas (true presence rate). A reason for this may be that the model sometimes misinterpreted acacia shrubs present in invaded areas as *Prosopis*; otherwise the unbalanced of sample size between presence and absence doesn't affect the quality the output (Jiménez‐Valverde & Lobo, 2006) as long as enough sample size were used from each group.

Machine learning algorithms have attracted significant attention in the modeling community. First, shallow Neural Networks (NN) attracted a lot of attention and were widely applied to many different research problems (Zhou et al., 2015). In the remote sensing community, the DNN was soon followed by other MLAs: the GBM‐BRT, the SVM, and the RF, which provided better results both in regression and classification (Ashby et al., 2017; Pal & Mather, 2005, 2017). Our regression analyses in the present study indicated that the RF, the ensemble model, and the GBM outperformed the SVM. A similar finding was reported by Lorena et al. (2011), who compared the performances of the RF and the SVM in modeling the potential distribution of 35 species in Brazil. The study by Mi, Huettmann, Guo, Han, and Wen (2017) also indicated that the RF performed better than other algorithms tested to model crane species.

Our finding confirms that the RF is a suitable algorithm for fractional cover mapping of plant species. However, based on our experiences gained during this study five important points should be considered in order to achieve good results while applying the RF regression: (a) sufficient and well‐distributed field data samples should be collected in the study area; (b) the number of presence and absence field samples should be proportional to the shares of the study area where the species is present and absent, respectively; (c) the field data values for the dependent variable should be within the range of the expected prediction values, (d) as shown by previous study, the values of explanatory variables used for training need to represent the entire range of values present in the study area (Hengl et al., 2015), and (e) fine‐tuning of algorithm parameters and variable reduction are recommended for improved model fitness and better regression outputs.

## 5 CONCLUSIONS

Fine‐scaled fractional cover maps of IAPS are a key requisite for estimating the environmental and socio‐economic impacts of IAPS and for designing spatially explicit management strategies. Our findings show that the RF regression is outperformed other algorithms and is a suitable for mapping the fractional cover of species distribution in agro‐climatic contexts similar to those of the Afar Region. While the GBM and the SVM achieved only slightly less accurate results, the GLM, the GBM‐BRT, and the DNN did not perform well when looking at sensitivity, specificity, kappa, and the AUC. Nevertheless, performances of MLAs might be different if a much larger amount of data (i.e., predictor variables) is used, or if less training data is available or if the study is done in a different agro‐ecological context. For this reason, we recommend evaluating the performances of two or more algorithms regarding the specific tasks required and the specific environmental settings prevailing in the context of plant species distributions.

## CONFLICTS OF INTEREST

The authors declare no conflict of interest.

## AUTHOR CONTRIBUTION

HS designed the sampling procedure, carried out the survey, and performed the modeling and the calculations; WB and SE provided technical advice; and all authors contributed to the writing of the manuscript.

## DATA AVAILABILITY

The dataset will be archived in an appropriate public archive.
