# Supplementary material for: Performances of machine learning algorithms for mapping fractional cover of an invasive plant species in a dryland ecosystem
Source: Ecol Evol. 2019 Feb 12;9(5):2562–74. doi: 10.1002/ece3.4919 (PMC6405495; doi:10.1002/ece3.4919)
Supplement: Supplementary file 1 [file ECE3-9-2562-s001.docx]

**Table S1:** Selected and fine-tuned parameter settings and used R packages for all models stability. Model abbreviations: generalized linear model (GLM), two different implementations of gradient boosting machines (GBM and GBM-BRT), random forest (RF), support vector machine (SVM), deep learning neural network (DNN), ensemble model (ENS). For detailed information of functions, arguments and their meaning we refer to the documentation of the respective R packages.

| **Model** | **Parameter settings** | **Selected parameter setting for parameters where several values were tested** | **Evaluation method of model stability** | **R-Packages** |
| --- | --- | --- | --- | --- |
| GLM | method = “cv”; number = 10;  family = binomial;  method = “glmStepAIC” |  | Step-AIC, automatic variable reduction, p-values | glmnet, rgdal, MASS |
| GBM | family = “ Gaussian”; method=“cv”; number =10; iterations=3,5,10,15;  method = “gbm”; bag.fraction=0.5;  ntrees=seq (50, 500, by = 50);  interaction depth=3; minobsinnode=100 | repeats = 10  ntrees = 500 | 10-fold cross-validation | devtool, dismo; Raster, caret; gbm, plyr |
| GMB-BRT | family = “bernoulli”; tree complexity = 5  learning rate = 0.01, 0.005;  bag fraction = 0.5; n-folds = 10;  ntrees = 50; max. trees = 10000  tolerance method = “auto”  tolerance = 0.001; all other settings were set to default values | learning rate = 0.005 | 10-fold cross-validation | dismo, gbm,  MASS |
| DNN | method = “cv”; number = 10  method = “dnn”; repeats = 5, 10, 15  all other settings were set to default values | repeats = 10 | 10-fold cross-validation | caret, raster, nnet, deepnet; devtools |
| RF | ntrees = 1000, 5000; method = “oob”; keep forest = TRUE; importance = TRUE; method = “rf’; all other settings were set to default values | ntrees = 5000 | 10-fold cross-validation  (also used for parameter tuning) | caret, rpart, devtools, raster, randomForest |
| SVM | SVM-Type=C-classification; SVM-Kernel = radial, linear, polynomial, sigmoid  cost = 1, 50,100, 150; gamma =0.1, 0.5, 1.0, 1.5 | kernel=radial  cost function=100  gamma=1 | 10-fold cross-validation (also used for parameter tuning) | libsvm, e1071, kernlab, rpart  dismo, rgdal |
| ENS | family = binomial; number = 10  repeats = 3,5, 10,15; method = "glmnet"; weighting = equal | repeats =10 | 10-fold cross-validation  AUC | devtools  caret, doParallel  raster, glmnet |

**Table S2:** Contributions and significances of twelve variables in the GLM

| Coefficients | Estimate | Standard error | Z- value | P-value (>\|z\|) |
| --- | --- | --- | --- | --- |
| (Intercept) | 1.96E+02 | 2.37E+01 | 8.274 | < 0.001 |
| DistRiver | -1.25E-05 | 7.17E-06 | -1.737 | 0.082 |
| DistVillage | -1.45E-04 | 2.43E-05 | -5.955 | < 0.001*** |
| Elevation | -6.20E-03 | 6.83E-04 | -9.071 | < 0.001*** |
| Landform | -2.40E-02 | 1.49E-02 | -1.611 | 0.1075 |
| LSTn | -6.55E+03 | 7.82E+02 | -8.374 | < 0.001*** |
| LTSd | 1.42E+03 | 2.08E+02 | 6.842 | < 0.001*** |
| NDVI | 1.34E+01 | 1.05E+00 | 12.731 | < 0.001*** |
| NIR | -1.77E+01 | 3.44E+00 | -5.143 | < 0.001*** |
| PAN | 9.953e+00 | 1.793e+00 | 5.551 | < 0.001*** |
| Rain | -2.18E-03 | 5.56E-04 | -3.923 | < 0.001*** |
| Red | 5.40E+00 | 1.96E+00 | 2.757 | < 0.01** |
| Temp | -1.55E-01 | 4.29E-02 | -3.601 | < 0.001*** |

df: 2086, and AIC: 1479.5; No. of Fisher Scoring iterations: 5;

**Model Parameter Settings for GBM-BRT**

In the GBM-BRT model, both the optimal number of trees and the model’s performance were affected by the learning rate. In a first scenario, we used a learning rate of 0.01 with 3100 trees; in a second scenario, we halved the learning rate (lr) to 0.005, which increased the optimal number of trees to 6050; and in a third scenario, with a lr of 0.0025, the optimal number of trees rose to 10,000; but comparing the three scenarios, the second one resulted in a more stabilized model and a more accurate output (Figure 2) than the other two. Both lr values of 0.005 and 0.0025 resulted in an AUC of 0.945, but the cross-validation correlation was smaller for 0.0025 (corr = 0.733) than for 0.005 lr (corr =0.741). Hence, the best fit was found at a learning rate of 0.005.


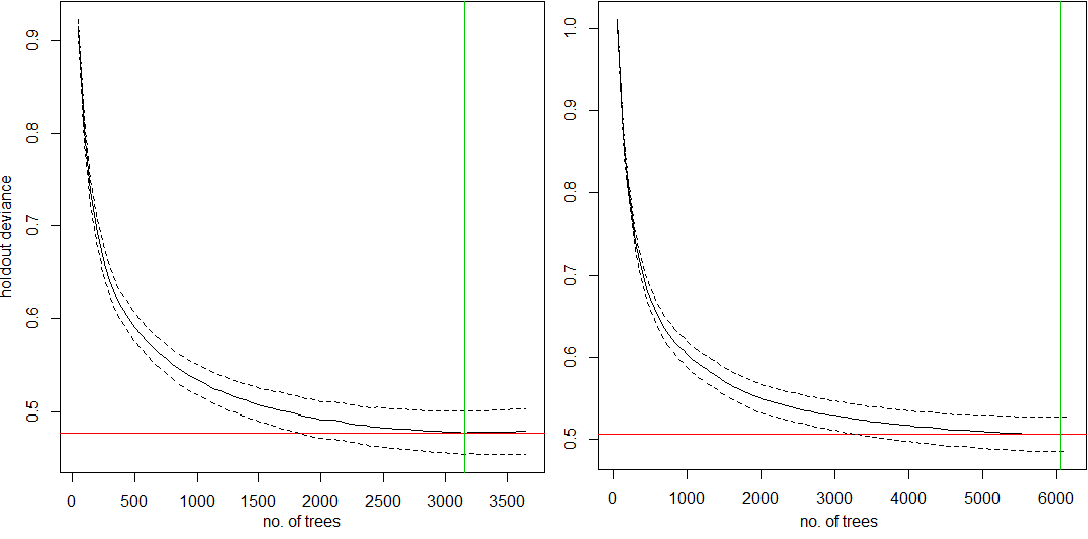


**Figure S1:** Optimal numbers of trees for a learning rate (lr) of 0.01 at 3100 trees (left) and 0.005 at 6050 trees (right) in the model based on gradient boosting machine (GBM-BRT). The solid line represents the mean predictive defiance; the dotted lines represent ± one standard error, measured on the excluded folds of the 10-fold cross validation. The minimum of the mean (red line) and the number of trees at which that occurs (green line) are indicted.

**
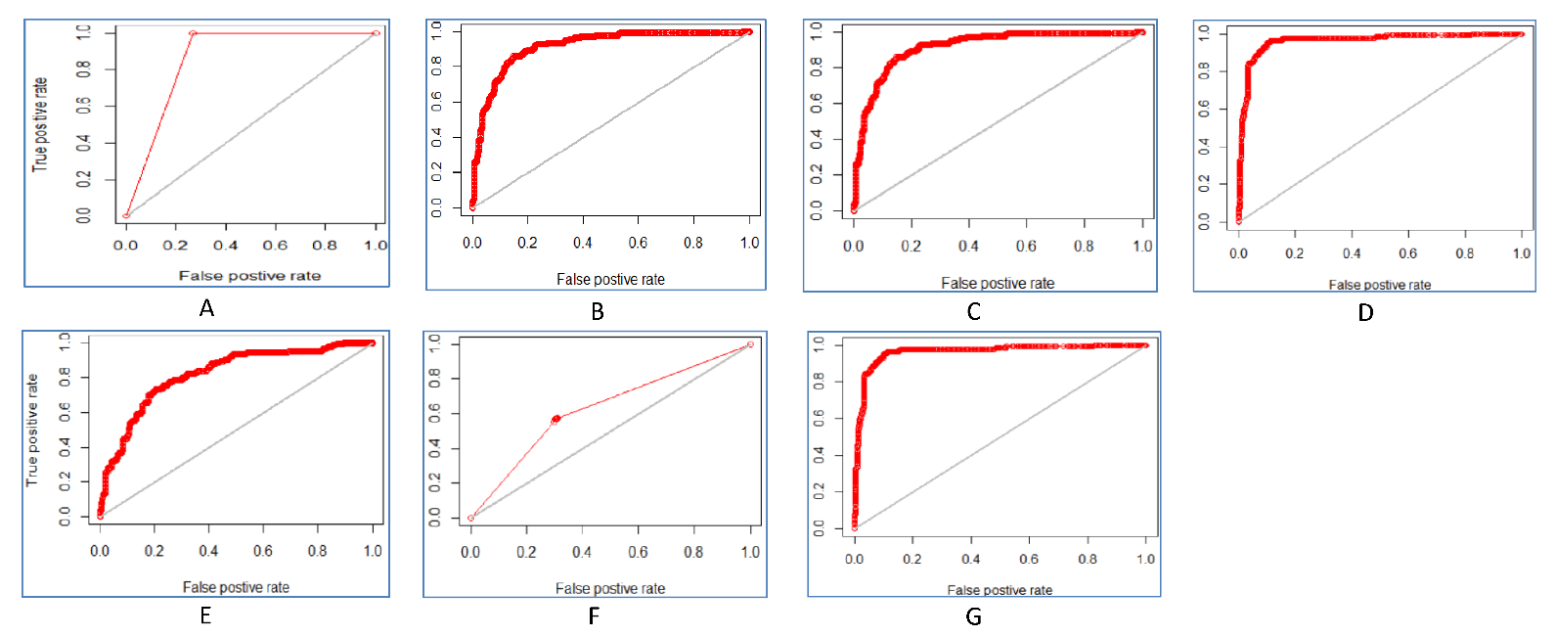
**

**Figure S2:** Area under the curve (AUC) for the various models: (A) generalized linear model (GLM) with AUC = 0.852; (B) gradient boosting machine (GBM) with AUC = 0.944, (C) gradient boosting machine using boosted regression trees package (GBM-BRT) with AUC = 0.945; (D) random forest (RF) with AUC = 0.971; (E) support vector machine (SVM) with AUC = 0.866; (F) deep neural network (DNN) with AUC = 0.595; and (G) ensemble model (ENS) with AUC = 0.962.
